# The impact of crystalloidal and colloidal infusion preparations on coronary vascular integrity, interstitial oedema and cardiac performance in isolated hearts

**DOI:** 10.1186/cc12898

**Published:** 2013-09-14

**Authors:** York A Zausig, Daniel Chappell, Bernhard F Becker, Daniel Potschka, Hendrik Busse, Kathrin Nixdorf, Diane Bitzinger, Barbara Jacob, Matthias Jacob

**Affiliations:** 1 Department of Anaesthesiology, University Hospital Regensburg, Franz-Josef-Strauß-Allee 11, 93053 Regensburg, Germany; 2 Department of Anaesthesiology, University Hospital Munich, Nussbaumstr. 20, 80336 Munich, Germany; 3 Walter-Brendel-Center of Experimental Medicine, University of Munich, Schillerstr. 44, 80336 Munich, Germany

## Abstract

**Introduction:**

Recent data suggested an interaction between plasma constituents and the endothelial glycocalyx to be relevant for vascular barrier function. This might be negatively influenced by infusion solutions, depending on ionic composition, pH and binding properties. The present study evaluated such an influence of current artificial preparations.

**Methods:**

Isolated guinea pig hearts were prepared in a modified Langendorff mode and perfused with Krebs-Henseleit buffer augmented with 1g% human albumin. After equilibration the perfusion was switched to replacement of one half buffer by either isotonic saline (NaCl), ringer's acetate (Ri-Ac), 6% and 10% hydroxyethyl starch (6% and 10% HES, resp.), or 4% gelatine (Gel), the artificial colloids having been prepared in balanced solution. We analysed glycocalyx shedding, functional integrity of the vascular barrier and heart performance.

**Results:**

While glycocalyx shedding was not observed, diluting albumin concentration towards 0.5g% by artificial solutions was associated with a marked functional breakdown of vascular barrier competence. This effect was biggest with isotonic saline and significantly attenuated with artificial colloids, the difference in the pressure dependent transvascular fluid filtration (basal vs. during infusion in groups NaCl, Ri-Ac, 6% HES, 10% HES and Gel, *n *= 6 each) being 0.31 ± 0.03 vs. 1.00 ± 0.04; 0.27 ± 0.03 vs. 0.81 ± 0.03; 0.29 ± 0.03 vs. 0.68 ± 0.02; 0.32 ± 0.03 vs. 0.59 ± 0.08 and 0.31 ± 0.04 vs. 0.61 ± 0.03 g/5min, respectively. Heart performance was directly related to pH value (7.38 ± 0.06, 7.33 ± 0.03, 7.14 ± 0.04, 7.08 ± 0.04, 7.25 ± 0.03), the change in the rate pressure product being 21,702 ± 1969 vs. 21,291 ± 2,552; 22,098 ± 2,115 vs. 14,114 ± 3,386; 20,897 ± 2,083 vs. 10,671 ± 1,948; 21,822 ± 2,470 vs. 10,047 ± 2,320 and 20,955 ± 2,296 vs. 15,951 ± 2,755 mmHg × bpm, respectively.

**Conclusions:**

It appears important to maintain the pH value within a physiological range to maintain optimal myocardial contractility. Using colloids prepared in calcium-containing, balanced solutions for volume replacement therapy may attenuate the breakdown of vascular barrier competence in the critically ill.

## Introduction

For over 100 years vascular barrier competence was generally acknowledged to be sufficiently explained by the historical principle of Ernest Starling [1]. This stipulated an inwardly directed oncotic gradient between an interstitial space presumably low in protein and the protein rich plasma with percentage albumin at concentrations of around 4 g% as main constituent. This gradient was considered to keep the compartments in balance, despite an intravascular hydrostatic pressure, which forces fluids and solutes outwards. The therapeutical target to prevent interstitial oedema whilst maintaining cardiac preload in the face of an unaffected endothelial cell line would have been, according to this model, to merely maintain the oncotic plasma pressure. The practical answer was the intravenous infusion of isooncotic colloids prepared in isotonic saline, irrespective of their binding properties for electrolytes and membrane coatings.

The last decade, however, has brought increasing evidence that things might not be that easy. Various experimental models showed that the interstitial oncotic pressure in most organs is far from zero and, surprisingly, does not relevantly influence transvascular filtration behaviour [2]. This brought attention to the endothelial glycocalyx, a negatively charged layer of proteoglycans and glycosaminoglycans, now identified as an important part of vascular barrier competence [3]. Due to its special biophysical and biochemical properties, the glycocalyx binds plasma constituents, forming the endothelial surface layer with a functional thickness of more than 1 µm [4]. It is only the oncotic gradient across this layer, that is, between the protein-loaded glycocalyx and a small space low in protein directly beneath, but completely at the luminal side of the anatomical vessel wall, that helps to limit hydrostatically driven outflow of plasma constituents in high-pressure segments of the circulation [2]. In addition, the endothelial surface layer plays an important role for shear stress transduction to the endothelial cells and also for generating an anti-inflammatory, anti-thrombotic and anti-adhesive vascular surface by harbouring adhesion molecules [5]. The fragile glycocalyx, however, can be degraded in various pathophysiological situations such as ischaemia/reperfusion, sepsis, hyperglycaemia, trauma or diabetes [6,7].

The integrity of the endothelial surface layer, which seems to be strongly related to oedema formation and cardiac performance [6], is strongly dependent on sufficient concentrations of suitable plasma constituents [8]. Moreover, the binding properties of both glycocalyx and plasma proteins should depend on plasma pH. Thus, acid-base chemistry should be considered when clinicians assess whether the composition of infusion preparations is adequate. Furthermore, some constituents of these preparations might disrupt the competence of the endothelial surface layer.

The present study investigated the influence of commercially available crystalloidal and colloidal infusion preparations on coronary vascular integrity, interstitial oedema and cardiac performance of isolated guinea-pig hearts. It extends the insight into what actually happens to microcirculation when diluting natural plasma constituents with artificial substitutes beyond a critical border. Therefore, these data might already help us today to rationally select a substitution solution for a critically ill patient, even in the absence of outcome-based evidence. Beyond that, such basic data may help to sharpen the focus of future developments in the field.

## Materials and methods

The investigation conformed to the Guide for the Care and Use of Laboratory Animals published by the US National Institutes of Health (NIH Publication No. 85-23, revised 1996) and was approved by - and licensure of the investigator obtained from - the Government of Upper Bavaria (Regierung von Oberbayern file No. 209.1/211-2531.3-3/99).

**Figure 1:**
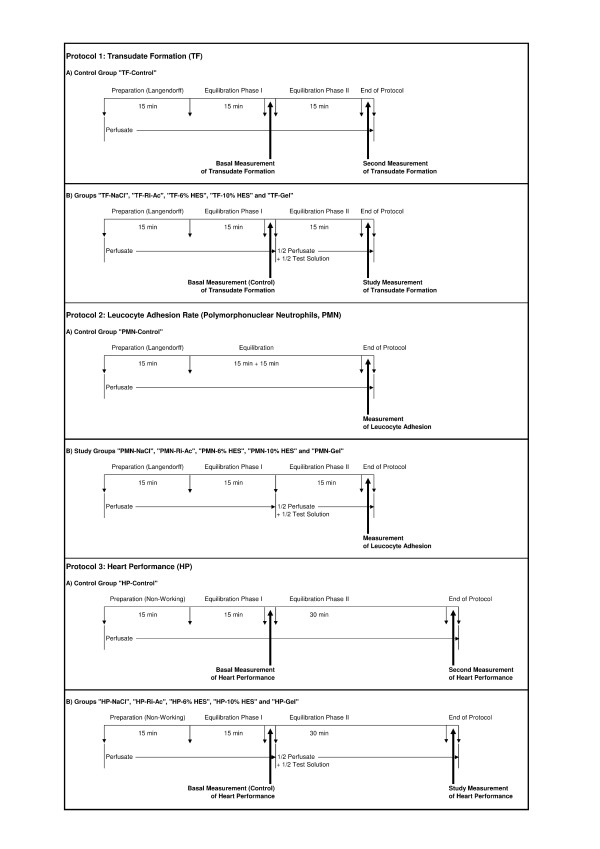
**Experimental protocols**. Protocol 1: transudate formation (TF). Sample generation for assessing transudate formation and glycocalyx shedding in control (TF-Control) and study groups (TF-NaCl, TF-Ri-Ac, TF-6% HES, TF-10% HES, and TF-Gel). The same hearts were used to determine interstitial water content after the protocol, and in two additional cases in each group, for electron microscopy evaluation. Protocol 2: polymorphonuclear neutrophils (PMN). The intracoronary adhesion rates of PMN were determined in control (PMN-Control) and study groups (PMN-NaCl, PMN-Ri-Ac, PMN-6% HES, PMN-10% HES, and PMN-Gel) at the time indicated. Application and evaluation followed the detailed description given within the running text. Protocol 3: heart performance (HP). The determination of heart performance occurred in control (HP-Control) and study groups (HP-NaCl, HP-Ri-Ac, HP-6% HES, HP-10% HES, and HP-Gel) at the times indicated. For details, see Materials and methods.

Hearts were prepared in a non-working Langendorff mode comparing basal conditions to intracoronary infusion of different artificial solutions (details see below). The unblinded study was arranged in three steps. Protocol 1 (Figure 1) assessed transvascular filtration immediately before and during infusion of the respective test solution, as well as interstitial water content at the end of protocol. Also, quantitative and qualitative glycocalyx shedding was determined. According to protocol 2 (Figure 1), human polymorphonuclear neutrophilic granulocytes (PMN) were infused intracoronarily and their adhesion rate was assessed. Protocol 3 (Figure 1) compared changes of heart performance and ionic and pH compositions when infusing the different solutions. The normal ranges for electrolytes, bicarbonate, phosphate and lactate in human plasma, in comparison to the composition of the tested crystalloidal and colloidal solutions, are presented in Table 1.

**Table 1:** Normal ranges of electrolyte, bicarbonate, phosphate and lactate in comparison to the composition of the studied solutions

	Plasma	NaCl	Ri-Ac	6% HES	10% HES	Gel	Unit of measurement
**Kations**							
Sodium	137 to 147	154	130	130	130	144	mmol/l
Potassium	3.5 to 5.5	NA	.5.4	.5.5	.5.5	5	mmol/l
Magnesium	0.8 to1.0	NA	1	1	1	.1.5	mmol/l
Calcium	2.2 to 2.6	NA	0.9	1	1	.2.5	mmol/l
**Anions**							
Chloride	95 to 110	154	112	112.5	112.5	103	mmol/l
Bicarbonate	23 to 25	NA	NA	NA	NA	NA	mmol/l
Phosphate	0.8 to 1.6	NA	NA	NA	NA	NA	mmol/l
Acetate	NA	NA	27	27	27	27	mmol/l
Lactate	0.0 to 1.0	NA	NA	NA	NA	NA	mmol/l
**Colloids**							
**HES **	NA	NA	NA	6	10	NA	g%
Gelatine	NA	NA	NA	NA	NA	4	g%
Theoretical osmolality	280 to 300	308	276	277	277	283	mosmol/kg H_2_O

NaCl, isotonic saline; Ri-Ac, ringer's acetate; 6% and 10% HES: 6% and 10% hydroxyethyl starch in Ringer's acetate. NA (not applicable).

### Heart preparation and perfusion

#### Basal preparations (protocols 1 and 2) (Figure 1)

Guinea pigs (male; weight 300 to 400 g) were stunned by neck dislocation, and immediately after opening of the thorax had their hearts arrested with ice-cold isotonic saline. The aorta was cannulated and the coronary arteries were reperfused *in situ *at a constant flow rate of 6 ml/minute (mean 37 ± SD 0.5°C, pH 7.40 ± 0.05). The perfusate was administered by a peristaltic pump (Type: MS-1 REGLO/8-160; Ismatec™ SA Laboratoriumstechnik, Glattbrugg-Zürich, Switzerland), the perfusion pressure being continuously recorded with a pressure transducer (FMI GmbH, Engelsbach, Germany). An initial perfusion pressure above 80 cm H_2_O predicted heart failure and led to exclusion. Hearts were removed from the thorax and preparation was finalised by ligating the caval and azygos veins and inserting a cannula into the pulmonary artery [9,10].

#### Additional preparations (protocol 3) (Figure 1)

A thin, saline-filled latex balloon (Hugo Sachs Electronic KG, March-Hugstetten, Germany) was inserted into the left ventricle and attached via a metal cannula to a pressure transducer (Isotec, Hugo Sachs Electronic KG) enabling measurement of isovolumetric systolic left ventricular pressure (LVP) development.

The initial intracoronary perfusion was carried out using a modified Krebs-Henseleit buffer (KHB, composition: 116mM NaCl, 23 mM NaHCO_3_, 3.6 mM KCl, 1.16 mM KH_2_PO_4_, 1.25 mM CaCl_2_, 0.58 mM MgSO_4_, 5.4 mM glucose, 0.3 mM pyruvate, and 2.8 U/l insulin, gassed with 94.5% O_2 _and 5.5% CO_2_) augmented with 1 g% human albumin from the onset of preparation. Electrolytes, pH and oxygen tension (Gem Premier 4000, Il GmbH, Kirchheim, Germany) and osmolality (Advanced Osmometer Model 2000, Advanced Instruments, Norwood, MA, USA) were measured.

### Experimental protocols

All hearts were randomly assigned to the respective groups before starting the preparation.

### Protocol 1 (Figure 1): transudate formation, glycocalyx shedding and interstitial water content (TF)

#### Transudate formation

Transudate filtered from the coronary system was collected on the epicardial surface in timed aliquots and quantified while the coronary venous effluent was collected from the pulmonary artery. Following preparation and the first equilibration phase in a dry warming chamber, the basal measurement of transudate formation was performed between minutes 25 and 30. In the control group (TF-Control, *n *= 6, protocol 1A) the perfusion continued for another 15 minutes before a second collection was carried out (minutes 45 to 50). In the study groups (TF-NaCl, TF-Ri-Ac, TF-6% HES, TF-10% HES and TF-Gel, *n *= 6 each, protocol 1B), the perfusion was switched after 15 minutes to a 1:1 mixture of buffer and the respective test solution following the basal measurement of transudate formation. The final determination was performed between minutes 30 and 45 following a second equilibration interval.

#### Glycocalyx shedding

Samples of coronary effluent were obtained from the same hearts after the first equilibration phase and again at the end of the second equilibration phase, each time for 3 minutes, and were stored at -20°C. The concentration of syndecan-1 (CD 138), a main component of the glycocalyx, was determined using an enzyme-linked immunosorbent assay (Diaclone Research, Besancon, France) as described before [11].

#### Tissue water content

At the end of the protocol, oedema formation was assessed in each heart by measuring ventricular wet weight (at once) and dry weight (after 24 h at 60°C). With interstitial oedema formation the quotient - wet weight × dry weight^-1 ^- increases *in vivo *from a mean value of 4.76 [8].

#### Electron microscopy

Two additional hearts per group were treated as described in protocol 1 for the TF-Control, TF-NaCl, TF-Ri-Ac, TF-6% HES, TF-10% HES and TF-Gel groups, but were prepared for electron microscopy (EM) as described previously [4] to qualitatively screen for morphological correlates of infusion-related glycocalyx shedding

### Protocol 2 (Figure 1): adhesion of polymorphonuclear neutrophils

Following preparation and the first equilibration phase the Langendorff-perfusion continued for another 15 minutes in the control group (PMN-Control, *n *= 6, protocol 2A) before application of PMN. In the study groups (PMN-NaCl, PMN-Ri-Ac, PMN-6% HES, PMN-10% HES and PMN-Gel, *n *= 6 each, protocol 2B), the perfusion mode switched to a 1:1 mixture of buffer and the respective test solution after the first equilibration interval. A second interval between minutes 30 and 45 preceded the assessment of PMN adhesion under the respective study conditions.

PMN of guinea pigs and humans show a quantitatively similar degree of adhesion in our model [12]. Therefore, the latter were used to substantially reduce the total number of animals needed. Preparation, intracoronary application and counting of non-adherent human PMN, as well as calculation of their adhesion rate were carried out as described previously [12]. Under basal conditions intracoronary retention has been shown to be 21.6 ± 2.1% [12]. Experimental shedding of the glycocalyx by intracoronary application of heparinase almost doubled this adhesion rate to 37.3 ± 2.0% [4,13].

### Protocol 3: heart performance (HP) (Figure 1)

For assessment of ventricular pressure development, balloon volume was adjusted to an initial diastolic LVP of 0 mmHg during the control period. Thus, any increase in diastolic LVP reflected an increase in left ventricular wall stiffness or diastolic contracture. The rate pressure product (RPP) was calculated as follows [14]:

$$\text{RPP}=\left(\text{Left}\;\text{ventricular}\;\text{systolic}\;\text{pressure}-\text{Left}\;\text{ventricular}\;\text{diastolic}\;\text{pressure}\right)\times \text{HR}$$

Following preparation and equilibration phase I, baseline values of HR, LVP and RPP were obtained. After that, basal perfusion was continued (HP-Control, *n *= 6, protocol 3A) or switched to the 1:1 mixture of buffer and the respective test solution (HP-NaCl, HP-Ri-Ac, HP-6% HES, HP-10% HES and HP-Gel, *n *= 6 each, protocol 3B). HP was re-assessed following equilibration phase II.

### Statistical analysis

The measured data are presented as mean ± SD, with n indicating the number of experiments.

To calculate the number of hearts needed, we performed a power calculation with G*Power 3.1 according to Faul *et al. *[15]. With *n *= 6, alpha-level of 0.05 and an assumed effect size

*d *>1, we expected a power level of 95% for the pairwise *t*-test with matched pairs. This number would also fit analyses of variance (ANOVA) with six groups and comparable values. The data for the effect size calculation being more than 1SD were based on previous studies [8]. Comparisons were made using the Student *t*-test for paired samples. For multiple comparisons, ANOVA was performed with the Bonferroni correction. In the case of unequal variances (group TF-Control and TF-NaCl), identified by the Levene test for homogeneity of variances, Dunnett's T3-test was performed. A *P*-value less than 0.05 was considered significant. The statistical software used to conduct the analyses was SPSS 16 (SPSS Inc., Chicago, IL, USA).

## Results

**Figure 2:**
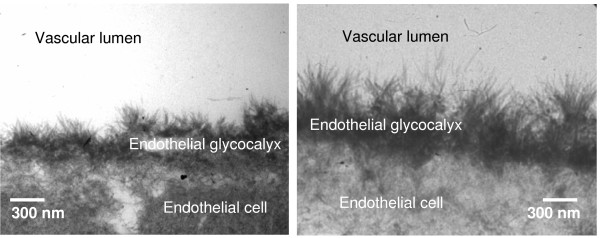
**Endothelial glycocalyx**. Exemplary electron microscopy picture of an intact endothelial glycocalyx after perfusion under control conditions (*TF-Control*, left panel) and after intracoronary infusion of 6% hydroxyethyl starch (*TF-6% HES*, right panel).

Electron microscopy of exemplary hearts from the control and the 6% HES group showed a tight glycocalyx at the endothelial surface at the end of perfusion protocols (Figure 2). Because no morphological differences were evident, tissue of hearts from other groups was not further investigated at this stage.

**Figure 3:**
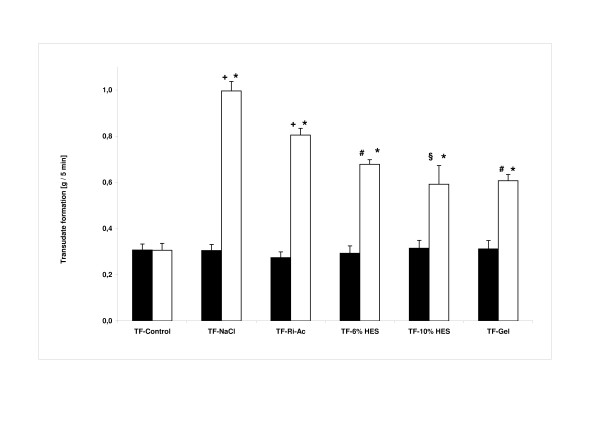
**Vascular integrity**. Transudate formation (all data expressed as mean ± SD; *n *= 6 for each group). Filled bars, basal measurement; open bars, second or study measurement. There were no significant differences among the basal measurements of all groups (analysis of variance (ANOVA): df = 5; *F *= 0.444; *P *= 0.814). *P <0.05 basal measurement versus study measurement (Student's paired *t*-test); ^+^*P *<0.05 versus all other basal and second/study measurements; ^#^*P *<0.05 versus all other basal and second/study measurements except study measurement of TF-10% HES; ^§^*P *<0.05 versus all other basal and second/study measurements except TF-6% HES and TF-Gel (ANOVA: df = 5; *F *= 172,644; *P *= 0.000; pairwise post hoc test performed with Dunnett´s T3-test).

Transudate formation and parameters related to HP showed no significant intergroup differences under basal conditions or between basal and second measurements in the control groups, respectively. However, a significant increase in transudate formation versus control conditions was observed in all study groups upon dilution of the modified, albumin-augmented KHB by artificial solutions (Figure 3). Vascular leakage was highest in the colloid-free groups TF-NaCl and TF-Ri-Ac, but significantly attenuated versus that in colloid groups TF-6% HES, TF-10% HES and TF-Gel. A complementary picture was found for the tissue water content, the wet-to-dry weight ratio for groups TF-NaCl, TF-Ri-Ac, TF-6% HES, TF-10% HES and TF-Gel (7.29 ± 0.03, 7.16 ± 0.36, 7.00 ± 0.21, 6.83 ± 0.09 and 6.45 ± 0.21, respectively) being significantly increased versus TF-Control (6.01 ± 0.03; *n *= 6 for each group; TF-Control versus TF-NaCl, TF-Ri-Ac, TF-6% HES and TF-10% HES: *P *<0.05; TF-10% HES versus TF-Control and TF-NaCl: *P *<0.05; TF-Gel versus TF-Control, TF-NaCl and TF-6% HES: *P *<0.05; TF-NaCl versus TF-Control, TF-10% HES and TF-Gel: *P *<0.05; ANOVA: df = 5; *F *= 25.380; *P *= 0.000; pairwise post hoc testing performed with Dunnett's T3-test).

PMN adhesion rates of groups PMN-Control, PMN-NaCl, PMN-Ri-Ac, PMN-6% HES, PMN-10% HES and PMN-Gel were 20.3 ± 0.6, 21.5 ± 0.5, 20.5 ± 1.0, 21.7 ± 1.2, 21.9 ± 1.1 and 20.6 ± 0.8%, respectively (*n *= 6 for each group). Though adhesion of PMN during infusion of 10% hydroxyethyl starch was significantly elevated versus control conditions (ANOVA: df = 5; *F *= 3.777; *P *= 0.009; pairwise post hoc test with Bonferroni correction: PNM-10% HES versus PNM-Control: *P *= 0.04; all others not significant), this effect was minimal. Shedding of syndecan-1 did not significantly differ among any of the investigated groups (not shown).

Substituting one half of the KHB by 6% HES (HP-6% HES), 10% HES (HP-10% HES) and HP-Ri-Ac decreased heart rate significantly compared with the respective basal value (174 ± 18 versus 221 ± 14, 176 ± 22 versus 216 ± 19 and 198 ± *25 *versus 222 ± 17 bpm), whereas no significant change was observed in group HP-Gel (222 ± 32 versus 217 ± 21 bpm). HP-NaCl, by contrast, showed a significant increase compared to the basal value (249 ± 14 versus 220 ± 21 bpm; *n *= 6 for each group; respective control versus study measurement of HP-NaCl, HP-Ri-Ac, HP-6% HES and HP-10% HES: *P *<0.05; HP-NaCl and HP-Gel versus HP-6% HES and HP-10% HES: *P *<0.05; ANOVA: df = 5; F = 19.458; *P *<0.001; pairwise post hoc testing performed with the Bonferroni correction).

LVP remained constant in control hearts (94 ± 12 versus 96 ± 11 mmHg), but decreased significantly during perfusion in all study groups versus the respective basal value (71 ± 13 versus 100 ± 9; 61 ± 7 versus 95 ± 11; 57 ± 11 versus 101 ± 10 and 72 ± 12 versus 97 ± 6 mmHg for groups HP-Ri-Ac, HP-6% HES, HP-10% HES and HP-Gel, respectively), except for HP-NaCl (86 ± 14 versus 99 ± 8 mmHg; *n *= 6 for each group; control versus HP-Ri-Ac, HP-6% HES, HP-10% HES and HP-Gel: *P *<0.05; HP-NaCl versus HP-10% HES: *P *<0.05; ANOVA: df = 5; *F *= 11.364; *P *<0.001; pairwise post hoc testing performed with the Bonferroni correction).

**Figure 4:**
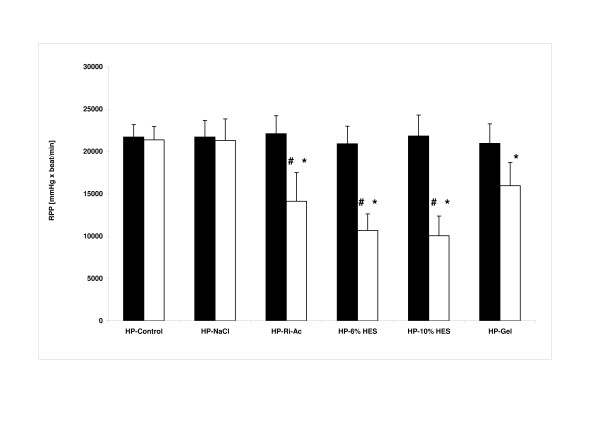
**Haemodynamic performance**. Rate-pressure product (RPP, all data expressed as mean ± SD; *n *= 6 for each group). Filled bars, basal measurement; open bars, second or study measurement. *P <0.05 versus Control; ^#^*P *<0.05 versus HP-NaCl (analysis of variance: df = 5; *F *= 29.361; *P *<0.001; pairwise post hoc testing performed with the Bonferroni correction).

These alterations resulted in significant decreases in the RPP compared to basal values (14,114 ± 3,386 versus 22,098 ± 2,115; 10,671 ± 1,948 versus 20,897 ± 2,083; 10,047 ± 2,320 versus 21,822 ± 2,470 and 15,951 ± 2,755 versus 20,955 ± 2,296 mmHg × bpm) (Figure 4).

The perfusate pH value was significantly reduced versus the modified KHB during perfusion with Ri-Ac (7.33 ± 0.03), 6% HES (7.14 ± 0.04), 10% HES (7.08 ± 0.04) and Gel (7.25 ± 0.03), all *P *<0.05, whereas NaCl did not cause a significant change (7.38 ± 0.06; *P *>0.05). During infusion of artificial solutions, electrolyte concentrations in the aortic inflow showed drug-dependent deviations. There was a reduction of sodium and calcium in all study groups with the exception of the NaCl group (Table 2). Potassium was only significantly decreased versus the basal perfusate in the NaCl group, whereas all other solutions led to a slight, albeit significant increase (*P *<0.05). Osmolality was not different among the groups (Table 2).

**Table 2:** Values measured within effective perfusate during study phase without (HP-Control) and with (HP-NaCl, HP-Ri-Ac, HP-6% HES, HP-10% HES and HP-Gel) infusion of respective preparation according to the study protocol

	HP-Control	HP-NaCl	HP-Ri-Ac	HP-6% HES	HP-10% HES	HP-Gel	Unit of measurement
**Sodium**	139 ± 1	148 ± 2*#	133 ± 2*	134 ± 4*	136 ± 2*	136 ± 2*	mmol/l
**Potassium**	.3.78 ± 0.04	.1.77 ± .0.05*#	.4.41 ± .0.09*	.4.46 ± .0.12*	.4.49 ± .0.09*	.4.21 ± .0.09*	mmol/l
**Calcium**	.1.86 ± 0.05	.0.97 ± .0.04*#	.1.33 ± .0.02*	.1.39 ± .0.05*	.1.44 ± .0.04*	.1.49 ± .0.11*	mmol/l
**Osmolarity**	269 ± 26	266 ± 34	256 ± 24	274 ± 16	276 ± 25	276 ± 5	mosmol/l
**pH**	.7.43 ± 0.04	.7.38 ± .0.06	.7.33 ± .0.03*	.7.14 ± .0.04*	.7.08 ± .0.04*	.7.25 ± .0.03*	

Electrolyte concentrations, osmolarity and pH in perfusate, assessed in the delivering lines of the HP groups under basal conditions (HP-Control) and after having switched to infusion mode using the respective study solutions (HP-NaCl, HP-Ri-Ac, HP-6% HES, HP-10% HES, and HP-Gel). All data expressed in mean ± SD; *n *= 6 for each group; *P <0.001 versus HP-Control; ^#^*P *<0.001 versus HP-Ri-Ac, HP-6% HES, HP-10% HES, and HP-Gel. HP, heart performance; NaCl, isotonic saline; Ri-Ac, ringer's acetate; 6% and 10% HES: 6% and 10% hydroxyethyl starch in Ringer's acetate.

## Discussion

Our study evaluated the impact of intracoronary infusion of commercially available crystalloidal and colloidal infusion preparations (test solutions, see Table 1) on coronary vascular integrity, HP, PMN adhesion and oedema formation of isolated guinea-pig hearts, with special focus on the endothelial glycocalyx. According to recent knowledge we augmented the basal Krebs-Henseleit perfusate with 1 g% human albumin to generate a sufficiently functional endothelial surface layer. As *in vivo*, this results from combining an intact endothelial glycocalyx with plasma proteins. Previous work [4,8] and preliminary experiments suggested that it might be difficult to generate marked disturbances of the endothelial surface layer when starting with physiological albumin concentrations around 4g%, because coronary washout requires too long [8]. Accordingly, by augmenting the buffer with merely 1g% human albumin we simulated conditions of a hypo-albuminaemic patient. Infusion of artificial solutions was simulated by replacing one half of the initial buffer, leading to a further reduction of the intracoronary albumin content towards 0.5g%. This protocol disclosed some marked effects.

Actual shedding of the endothelial glycocalyx was obviously not initiated by any solution or condition simulated, as demonstrated by no increase in the glycocalyx constituent syndecan-1 in coronary effluent, no morphological hint on the basis of electron microscopic examination, and no relevant increase in leukocyte adhesion. However there were some considerable functional defects of vascular competence. In particular, we observed an increase in transudate, the equivalent of lymph flow in the isolated heart model and a direct measure of vascular fluid leak. This was noted especially when using colloid-free infusion solutions, these obviously diluting the colloid content of the net perfusate below a critical level. Presumably, this pathological microvascular disturbance is reversible, because no overt damage to the glycocalyx was detected.

Interestingly, infusion of the non-balanced sodium chloride had, despite a physiological pH-value of the mixed perfusate, a significantly worse effect on vascular leak than infusion of the balanced Ringer's acetate solution, which led to a slight metabolic acidosis of the perfusate in our model. These effects on pH value, however, are totally contrary to those seen under clinical conditions, where the infusion of sodium chloride is associated with severe hyperchloraemic acidosis, which does not occur with balanced solutions [16]. The reason is that after intravenous infusion into the human circulation, the anion acetate is rapidly metabolised into bicarbonate, preventing acidosis. The isolated heart is obviously unable to metabolise acetate rapidly enough to prevent an artificial acidosis. Therefore, the anti-acid effect of balanced solutions might be underestimated in this *in-vitro *study. Isotonic saline, by contrast, is inert in terms of acid-base chemistry in our model, but causes severe hyperchloraemic acidosis *in vivo *[17]. Accordingly, it would seem there is an additional beneficial impact of balanced solutions on coronary vascular permeability, beyond pure osmotic and pH phenomena, most likely related to the ionic composition. The higher concentrations of potassium and free calcium ions would be the most likely ones. In particular, calcium should play an important role in this context, an ion that is not present in sodium chloride solution but in all other solutions tested here.

Both 6 and 10% hydroxyethyl starch and 4% gelatine are also based on balanced crystalloid and additionally, contain an artificial colloid. Although previous work suggests that hydroxyethyl starch should be inferior to human albumin in terms of interaction with the glycocalyx to form an endothelial surface layer, the present study applied the artificial colloids in combination with human albumin, thus imitating the scenario of clinical practice. As was to be expected, these preparations were able to maintain vascular barrier function much better than crystalloids [4]. Hypothetically, the mainly negative charges of gelatine, being a protein, could imitate the properties of natural plasma proteins much better than starch, a carbohydrate molecule derived from plants. Indeed, tissue oedema proved to be lower in hearts perfused with gelatine solution, suggestive of a tighter endothelial surface layer.

The *in-vitro *observations of HP display a direct relationship to the pH value of the coronary inflow: the more acidosis, the more decrease in LVP and HR. A tight control of acidosis might thus preserve myocardial functioning, which confirms the results of previous studies [17]. It has been shown that acidosis indirectly influences excitation-contraction coupling by decreasing the delivery of calcium to the myofilaments (for example, by inhibition of calcium current at the calcium channel or calcium release of the sacroplasmic reticulum), leading to depressed myocardial performance [18,19].

Additionally, reduction of the calcium concentration in the perfusate due to the addition of artificial solutions (Table 2) might have also affected left ventricular function. In isolated heart models, calcium - one of the key determinants of contractile function - shows a direct relationship with left ventricular developed pressure. It is of note that the HP-NaCl group was the only one with an increase in heart rate, possibly due to the reduced potassium concentration. This increase might have offset the effects of reduced pH and calcium concentration on left ventricular performance.

It is difficult to rule out any direct or indirect effects of the tested artificial colloids on the rate-pressure product. For example, there is a study showing that artificial colloids as additive to cardioplegic solution do have an influence on myocardial oedema development and preserve cardiac function [20]. However, these particular results pertain to ischaemia-reperfusion conditions and there was a different composition of the perfusion solution. Other data suggest that artificial colloids affect endothelial function by reducing endothelium-dependent relaxation and the endothelium-derived hyperpolarising factor [21]. This might interfere with coronary flow regulation, thereby indirectly influencing left ventricular function [22]. However, as we used constant flow perfusion to provide an additional element of constancy in our study, autoregulatory coronary flow mechanisms would be overridden and any such colloidal endothelial effects neutralised. As opposed to such vague possible actions of the different colloids, the changes in pH of the final perfusate mixtures are closely aligned to the observed functional changes on rate-pressure product (cf. Table 2 and Figure 4). There was no such association for the level of potassium.

An important limitation of our results is the use of red blood cell-free perfusates. This is associated with a low viscosity and a low CaO_2 _(PaO_2_/oxygen content) and leads to a higher coronary blood flow than *in vivo*, possibly associated with an increase of shear stress, and consequently an endothelial release of vasoactive substances [23]. Admixture of colloids will also influence viscosity and shear stress versus crystalloid buffers. To alleviate such influences, our perfusion was flow controlled, which additionally eliminated possible effects on flow leading to Gregg's phenomenon [24]. However, because red blood cells influence metabolic and acid-base balances, somewhat different results for the haemodynamic parameters cannot be ruled out in the event of admixture to whole blood [25].

## Conclusions

The presented study investigated the impact of intracoronary perfusion of crystalloidal and colloidal infusion preparations as used in the clinical setting in an isolated guinea-pig heart model. In no case was there evidence for rapid destruction of the endothelial glycocalyx. Nevertheless, the tested solutions produced a notable breakdown of vascular competence, which was most impressive when using isotonic saline and significantly attenuated when using colloidal infusions. Infringement of heart performance showed a correlation with a decreasing pH. Therefore, maintaining the pH value within a physiological range is important to preserve myocardial function. Prevention of oedema requires the use of colloids prepared in calcium-containing, balanced solutions for volume replacement therapy.

## Key points

• Isotonic saline in colloidal infusions shows notable breakdown of vascular competence.

• It appears important to maintain the pH value within a physiological range to maintain optimal myocardial contractility.

• Oedema formation may be limited by using colloids prepared in calcium-containing, balanced solutions for volume replacement therapy, instead of crystalloids.

## Abbreviations

EM: electron microscopy; 6% HES: 6% hydroxyethyl starch in Ringer's acetate (Vitafusal 6%, Serumwerk Bernburg AG, Bernburg, Germany); 10% HES: 10% hydroxyethyl starch in Ringer's acetate (Vitafusal 10%, Serumwerk Bernburg AG, Bernburg, Germany); Gel: 4% modified Gelatine in ringer's acetate (Gelafusal, Serumwerk Bernburg AG, Bernburg, Germany); HP: heart performance; HR: heart rate; KHB: Krebs-Henseleit buffer; LVP: isovolumetric systolic left ventricular pressure; NaCl: isotonic saline; PMN: human polymorphonuclear neutrophilic granulocytes; Ri-Ac: Ringer's acetate (Ringer-Acetat-Lösung Bernburg, Serumwerk Bernburg AG, Bernburg, Germany); RPP: rate-pressure product; TF: transudate formation.

## Competing interests

This study was performed using departmental research funding provided by the Bavarian government (Bayerisches Staatsministerium für Wissenschaft, Forschung und Kunst, München; Bavarian State Ministry of Science, Research and the Arts, Munich). In addition, an unrestricted grant was given to the Department of Anaesthesiology, University Hospital Munich by Serumwerk Bernburg AG (Bernburg, Germany). It was not linked to any influence on study design or manuscript approval by the company.

MJ has held lectures for and received grants from Baxter Deutschland GmbH (Unterschleißheim, Germany), B. Braun Melsungen AG (Melsungen, Germany), Fresenius Kabi Deutschland GmbH (Bad Homburg, Germany), Grifols (Barcelona, Spain), and Serumwerk Bernburg AG (Bernburg, Germany) and is a member of the Grifols Albumin Advisory Board. DC has held lectures for Fresenius Kabi Deutschland GmbH (Bad Homburg, Germany) and has received a research grant from Grifols (Barcelona, Spain). All other authors have no possible conflicts of interest.

## Authors' contributions

YAZ, DC and MJ initiated the study, developed the study protocol, analysed and interpreted the data and wrote the manuscript. BFB, DP, DB and HB performed the experiments, analysed and interpreted the data and coordinated the logistic part of the study. KN accomplished the electron microscopy. BJ performed the statistical analyses. All authors drafted the manuscript and revised it critically for important intellectual content and approved the final manuscript.
